# The cost-effectiveness of tumour-infiltrating lymphocyte cell therapy for advanced melanoma: a systematic review

**DOI:** 10.1186/s12885-026-15888-5

**Published:** 2026-03-20

**Authors:** Mengjun Wu, James Larkin, Andrew Furness, Charitini Stavropoulou

**Affiliations:** 1 School of Health and Medical Sciences, City St George’s, University of London, London, UK; 2 The Royal Marsden NHS Foundation Trust, London, UK

**Keywords:** Tumour-infiltrating lymphocytes, Standard treatment, Advanced melanoma, Economic evaluation, Systematic review

## Abstract

**Background:**

Adoptive cell therapy with tumour-infiltrating lymphocytes has been recognised as a safe and effective treatment for patients with advanced melanoma. Whether such a novel intervention is cost-effective remains unclear. The aim of this research is to evaluate the cost-effectiveness of this intervention for advanced melanoma by systematically reviewing the existing literature.

**Methods:**

Electronic databases (including MEDLINE, EMBASE, PubMed, Web of Science, Cochrane Trials, ClinicalTrials.gov, CINAHL, Academic Search Ultimate, Centre for Reviews and Dissemination’s National Health Service Economic Evaluation Database, and citations) were searched for published cost-effectiveness studies from 2000.

**Results:**

A total of two studies met the inclusion criteria of a full economic evaluation. According to both studies, this novel intervention is deemed as cost-effective for advanced melanoma compared to standard treatment ipilimumab in a Dutch and Danish setting. The finding supports reimbursement of this intervention and lead to its inclusion as treatment guidelines for patients with advanced melanoma.

**Conclusions:**

However, given the little research relating to its cost-effectiveness, further clinical trial investigations could be the next step of research in the UK and other European countries would be invaluable for these healthcare systems and their patients.

**Supplementary Information:**

The online version contains supplementary material available at 10.1186/s12885-026-15888-5.

## Introduction

Advanced melanoma is an aggressive malignancy associated with poor prognosis in the absence of effective therapeutic interventions. An estimate of 325,000 patients was diagnosed with melanoma worldwide in 2020, which led to 57,000 deaths [1]. Clinical outcomes in patients with advanced melanoma have been substantially improved following the introduction of targeted therapies and immune checkpoint inhibitors (ICI) in the last decades [2–3]. When first-line ICI treatment with anti-PD-1 antibodies in patients with unresectable stage IIIC-IV melanoma was ineffective, second-line treatment with ipilimumab (anti-CTLA-4 antibody) monotherapy or ipilimumab and nivolumab combination therapy offered modest response rates up to 13% and 31% respectively [4–5]. Furthermore, second-time treatment with BRAF/MEK has been observed to offer better response rate of 22%-57%, however this was typically a short-lived clinical benefit [6–7]. Therefore, given that a large proportion of patients do not respond or eventually relapse, there is a clear medical need for novel treatment modalities for patients with refractory unresectable stage IIIC-IV melanoma.

Adoptive cell therapy with tumour-infiltrating lymphocytes (TIL therapy) demonstrates promising antitumour activity in patients with advanced melanoma. TIL therapy has been recognised as a safe and effective treatment for patients with metastatic melanoma in multiple non-randomised single centre phase II clinical trials in the USA, Israel and Europe [8–13]. In a recent multicentre, open-label, randomised phase III clinical trial, the results have shown statistically significant and clinically relevant improved progression-free survival in patients with unresectable stage IIIC-IV melanoma [14]. Despite the evidence of its clinical effectiveness, little is known about the cost-effectiveness of TIL therapy amongst patients with advanced melanoma.

Here, we seek to systematically review and critically assess the evidence on the cost-effectiveness of TIL therapy for advanced melanoma.

## Methods

This review adheres to the Preferred Reporting Items for Systematic Reviews and Meta-Analyses 2020 (PRISMA) statement [15] and was registered on the PROSPERO database, International prospective register of systematic reviews (registration number: CRD42024610005), available from https://www.crd.york.ac.uk/prospero/display_record.php?ID=CRD42024610005.

### Search strategy

An extensive literature search was conducted using electronic databases that included MEDLINE, EMBASE, PubMed, Web of Science, Cochrane Trials, ClinicalTrials.gov, CINAHL, Academic Search Ultimate, Centre for Reviews and Dissemination’s National Health Service Economic Evaluation Database (NHS-EED) and citations. The search terms used were organised into three key concepts including (i) advanced melanoma (e.g., unresectable stage IIIC-IV or metastatic melanoma); (ii) tumour-infiltrating lymphocytes (TIL); and (iii) economic evaluation (e.g., cost-effectiveness analysis (CEA), cost-utility analysis (CUA) and cost-benefit analysis (CBA)). Having removed duplicate papers, we considered first the titles and abstracts and then full texts for inclusion/exclusion. Screening was undertaken by MW and doubled-checked by CS between 29th May and 12th June 2025, and 9th and 13th February 2026. Any variation in decisions was resolved by discussion between MW and CS.

Studies were included if they undertook a trial or model-based economic evaluation (i.e., two or more interventions considering costs and outcomes). This led to exclusion of studies if they report only clinical endpoints and do not investigate any economic outcomes. Furthermore, studies such as reviews, editorials, commentaries and methodological articles were also excluded. Studies published before 2000 and in languages other than English were excluded.

### Data extraction

Characteristics of the studies were extracted into a standardised table that was adapted from the review guideline for economic evaluations developed by the Joanna Briggs Institute [16]. The data extraction table included characteristics of the population, country, perspective, intervention, time horizon, type of economic evaluation (i.e., CEA, CUA or CBA), study design (i.e., trial or model-based), costs, outcome measures (e.g., QALYs), and cost-effectiveness results (e.g., the incremental cost-effectiveness ratio [ICER]). Data extraction was undertaken by MW and double-checked by CS, and disagreements were resolved by discussion between MW and CS.

### Data synthesis

Findings were synthesised qualitatively and presented as a narrative summary in conjunction with a tabular summary.

### Quality assessment

The Quality of Health Economic Studies Instrument (QHES) was used to assess the quality of included studies [17]. The instrument contains 16 yes or no questions and each question is rated based on its importance. All the points for the questions answered yes are then to be added to calculate the quality score of a study. To determine the quality the cutoff points are as follows: 0–24 (extremely poor quality); 25–49 (poor quality); 50–74 (fair quality); and 75–100 (high quality). Quality assessment was undertaken by MW and double-checked by CS, and disagreements were resolved by discussion between MW and CS.

## Results

**Fig. 1:**
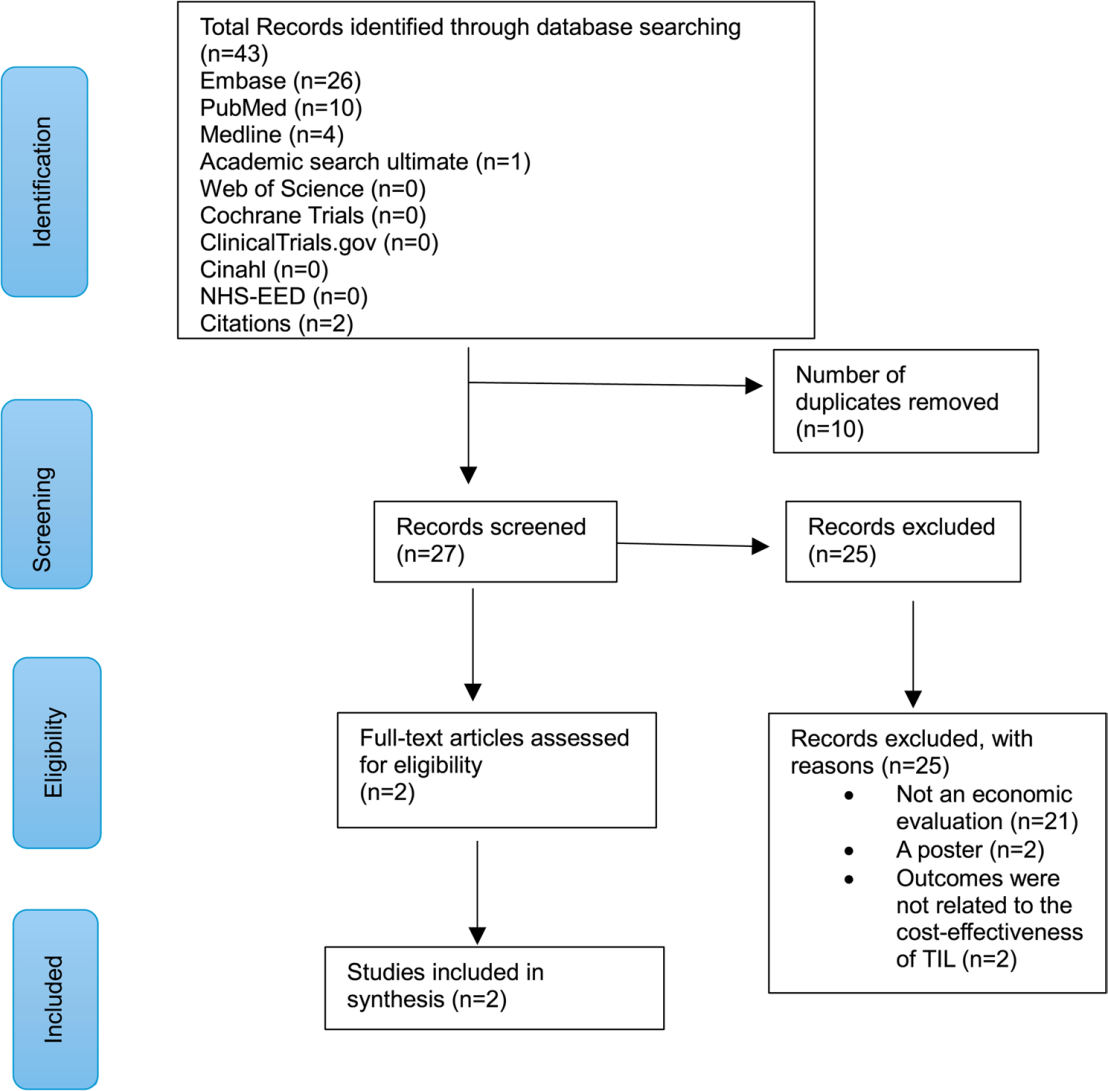
PRISMA flow diagram

Figure 1 presents the PRISMA flow diagram. 43 articles were identified through database searching, after removing duplicates, and screening titles, abstracts and full texts, two studies were finally included for data extraction and quality assessments. Basically, the 10 articles from PubMed were duplicate to the 10 articles out of 26 from Embase; the four articles from Medline were duplicate to the four articles out of 10 from PubMed; and the two citations were duplicate to the two articles out of 4 from Medline. Therefore, that left us with 26 articles from Embase and one article from Academic search ultimate for screening, and only two studies were included after screening. As an illustration, the search strategy for EMBASE is presented in Supplementary Table 1.

### Characteristics and main findings of included studies

Table 1 presents the characteristics and main findings of the included studies. There were two studies that evaluated the cost-effectiveness of TIL therapy versus ipilimumab in patients with unresectable stage IIIC or IV melanoma. One study was conducted in the Netherlands (*n* = 10), and the other in the Netherlands and Denmark (*n* = 168). The common primary method of economic evaluation used was construction of a Markov decision model with CUA in a Dutch setting. The second study further conducted a scenario analysis using a societal perspective in a Danish setting.

**Table 1:** The characteristics of included papers

^Lead author (year), country^	^Targeted disease^	^Population description^	^Intervention^	^Comparator^	^Evaluation^ ^type^	^Study design^	^Perspective, time horizon^	^Year of pricing, discount rates^	^Cost categories^	^Outcomes^	^Results ICERs^
^Retel and colleagues (2018) [18], the Netherlands^	^Advanced melanoma (metastatic melanoma, stage IV)^	^Adults starting at age 52^	^Tumour−infiltrating lymphocytes (TIL)^	^Ipilimumab^	^Markov decision model (CUA)^	^Pilot phase (*n*=10)^	^The Dutch health care system, lifetime horizon^	^2015, 4% on costs, 1.5% on effects^	^Intervention costs, healthcare costs^ ^TIL: €81,140^ ^Ipi: €94,705^	^QALYs^ ^TIL: 0.45^ ^Ipi: 0.38^	^TIL was more effective and less costly, 86% being cost effective, ICER: €80,000^
^ten Ham and colleagues (2024) [19], the Netherlands and Denmark^	^Unresectable stage IIIC−IV melanoma after failed first−line or second−line treatment^	^Patients with unresectable stage IIIC or stage IV melanoma^	^Tumour−infiltrating lymphocytes (TIL)^	^Ipilimumab^	^Markov decision model (CUA)^	^RCT (*n*=168)^	^The Dutch and Danish health care system, lifetime horizon^	^2012, 4% on costs, 1.5% on benefits in the Dutch setting; 2021, 3.5% on both costs and benefits in the Danish setting^	^Treatment costs,^ ^healthcare costs,^ ^societal costs^ ^Dutch^ ^TIL: €347,168^ ^Ipi: €433,634^ ^Danish^: ^TIL: €337,309^ ^Ipi: €436,135^	^QALYs^ ^TIL: 3.22^ ^Ipi: 2.28^	^TIL is cost−effective and cost−saving^ ^Dutch: 99% being cost effective,^ ^ICER: €80,000^ ^Danish: 99% being cost effective,^ ^ICER: €50,000^

The first study, by Retel and colleagues in 2018 [18], performed a cost-effectiveness analysis on TIL therapy versus ipilimumab for second-line treatment in advanced melanoma patients from a Dutch healthcare perspective over a lifetime horizon. A Markov decision model was employed to estimate the expect costs and outcomes for TIL therapy versus ipilimumab. Simulation was undertaken on a hypothetical cohort of 1,000 patients aged 52 with stage IV melanoma in the stable disease health state, which can develop into the progressive disease and death health states. TIL therapy was found to generate more QALYs at lower incremental costs compared to ipilimumab, and produced a dominant ICER (i.e., less costly and more effective) and had a probability of 86% being cost-effective at a willingness-to-pay threshold of €80,000. Nevertheless, given the substantial uncertainty with this first cost-effectiveness analysis, it was recommended that further exploration of the cost-effectiveness of TIL therapy would be best conducted in a randomised controlled trial.

The second study, by ten Ham and colleagues [19] produced the first cost-effectiveness analysis of TIL therapy based on the data from a multicentre, open-label, randomised controlled phase III trial [14] compared with ipilimumab in 168 patients with unresectable stage IIIC-IV melanoma after failed first-line or second-line treatment. In the Dutch setting, a Markov decision model was employed to simulate treatment sequence, including three mutually exclusive health states, namely progression-free survival, progressive disease and death. They also found that incremental QALYs were gained at lower costs, resulting in a dominant position for TIL therapy compared with ipilimumab. TIL therapy produced a probability of 99% being cost-effective at a willingness-to-pay threshold of €80,000. In the Danish setting, a scenario analysis was conducted using a societal perspective over a lifetime horizon. TIL therapy was also found in a dominant position with higher QALYs and lower total costs, with a probability of 99% being cost-effective at a threshold of €50,000. Overall, in both the Dutch and Danish setting, the likelihood of TIL therapy being cost-effective compared with ipilimumab is 99%.

### Quality assessment

The quality scores ranged from 97 to 100, which means both studies achieved high quality. Details of quality scores for each study are presented in Supplementary Table 2.

## Discussion

In March 2024, the US Food and Drug Administration approved the first TIL therapy in the world [20]. While this was a ‘long-awaited go-ahead’, the use of TIL therapy in clinical practice will depend not only on its effectiveness but also its cost-effectiveness [21]. This review provides a summary on economic evaluation studies of TIL therapy since 2000. Findings from the current review highlight that there has been little research relating to the cost-effectiveness of TIL therapy compared to standard treatment ipilimumab. Furthermore, the evidence is restricted in two high-income settings, the Netherlands and Denmark. Using constructed Markov decision model, both studies showed that TIL therapy was highly likely to be cost-effective compared to standard ipilimumab, providing some initial evidence for patients with advanced melanoma.

### Limited generalisability

Findings from two studies alone have limited generalisability. Both studies have been conducted in countries with universal healthcare systems and results may differ in different settings. Further studies focusing on different settings and populations will provide more evidence on the cost-effectiveness of this type of therapy.

There has also been substantial variability in the price of ipilimumab, which can have an impact on the transferability of the results. Ipilimumab is administered intravenously over a 90-minute period every three weeks for a total of four doses. In the UK, the price for ipilimumab is £3,750 per 50 mg vial, which bring the average cost per patient to £75,000 [22]. In the US, ipilimumab costs $7,308 per 50 mg vial bringing the average overall cost to $146,160 per patient [23], which is significantly higher than that in the UK. Among 14 European countries, the total ipilimumab costs range from €46,777 in Spain with high discounts to €110,000 in Lithuania [24].

### Uptake of TIL therapy

In addition to its cost, the uptake of TIL therapy will also depend on the access to TIL manufacturing and cellular therapy-capable healthcare facilities. Chen and colleagues noted TIL therapy faces challenges such as complex manufacturing processes [25], even though centralised TIL manufacturing platforms have been effective in producing consistent, scalable products at lower cost and with greater accessibility [26]. Yet it can be challenging to expand such centralised facilities globally to broaden patient access. Furthermore, production and logistical challenges may require attention and action in large scale implementation of TIL therapy due to its personalised nature. TIL therapy faces complex production process, involving extracting TILs from a patient’s tumour, expanding them ex vivo, and then infusing them back into the patient, which can take several weeks to months. Access to TIL therapy may be limited by geographic and logistical barriers, which can complicate the logistics of patient care and follow-up. These implementation issues need to be considered alongside methodological limitations of the cost-effectiveness analysis.

There are also potential impact and implications of ipilimumab coming off patent and the increasing availability of biosimilars [27]. The patent for ipilimumab expired in the US in March 2025 and expires in the EU by February 2026, which have created opportunities for biosimilar manufacturers to enter global markets. In turn, this would bring the prices of biosimilars down, and lower prices may reduce overall oncology expenditure, potentially creating financial budget for new innovative therapies. Moreover, the introduction of biosimilars would bring reduced financial barriers to immunotherapy and enable wider adoption of ipilimumab and nivolumab combination therapy. Lower prices would also bring broader access to first-line immunotherapy options, and possibly to checkpoint inhibitors by patients in underserved health systems. Overall, the impact of expiration of ipilimumab patents is far-reaching, including boosting competition, lowering costs significantly, and increasing patient access globally.

### Study limitations

There are some methodological limitations in the current studies reviewed. One notable methodological issue relates to the early stage of TIL production and other sources used to gather the survival data for TIL therapy in the Retel et al. study [18]. The survival data were pooled from two phase II studies which produced a substantial amount of uncertainty in the model-based economic evaluation. Uncertainty was however addressed using probabilistic sensitivity analyses to examine the reliability of cost-effectiveness inferences. Additionally, the cost estimation was based on a small sample of 10 patients, which may not provide sufficient variability to detect meaningful differences in costs, leading to limited statistical generalisability. The complexity of this early cost-effectiveness analysis of TIL therapy may not be captured in the limited sample size, making it difficult to draw broader conclusions.

The ten Ham et al. study [19], a multicentre, randomised, phase III clinical trial conducted in the Netherlands and Denmark, addressed the aforementioned data collection problems. However, the cost estimations in the trial employed activity-based costing approach. The clinical trial did reflect true production costs of TIL therapy, but did not include marketing costs, profit margins, and early investments, which would have produced an underestimation compared to a commercial setting [28]. Furthermore, extrapolating trial data beyond trial duration to a lifetime horizon would have made assumptions regarding future follow-up activities. The lifetime horizon assumes that the impact of TIL therapy will persist throughout the patient’s life. In other words, when extrapolating cohort experience into the future, assumptions would have been made about the continued efficacy of TIL therapy, and health outcomes and costs of care were projected forward with a discounting rate.

Notwithstanding this review identified a small number of studies that suggest TIL therapy is cost-effective, the scale-up of such an intervention requires further consideration. In ten Ham et al. study [19], the trial showed that after disease progression on TIL therapy, 11% of patients switched to ipilimumab and nivolumab combination therapy from the standard care of ipilimumab. Nevertheless, the cost-effectiveness of this comparator was not included, and no comparison was made between TIL therapy versus ipilimumab and nivolumab, even though this phase III trial cost-effectiveness analysis of TIL therapy made an impact on treatment guidelines in a Dutch and Danish setting. Neither of the two trials discussed the dropout rates, but trial-based studies may have high dropout rates, indicating problems with acceptability, adherence, and feasibility of TIL therapy. The QHES may have limitations given that the assessment questions have a focus on the key aspects of model-based economic evaluations. This may have led to higher quality scores for both studies, given the lack of an option to assign a middle score for each question in the QHES.

## Conclusion

In conclusion, evidence on the cost-effectiveness of TIL therapy for advanced melanoma is still very limited but suggests that it can be cost-effective, for patients with unresectable stage IIIC or IV melanoma. An important determinant will be the price of ipilimumab. Further clinical trial investigations of TIL therapy in other settings such as the UK and other European countries would shed more light in this area. Future trials investigating costs and survival, health-related quality of life of ipilimumab and TIL therapy would be valuable for further cost-effectiveness analyses in these settings.

## Supplementary Information

Supplementary Material 1.

## Data Availability

All data analysed during this study are included in its supplementary information files. Supplementary information: Supplementary Table (1) Search strategy for EMBASE; Supplementary Table (2) Quality assessment result of included studies using the QHES; Supplementary Table (3) Completed PRISMA Checklist.
