# Supplementary material for: The cost-effectiveness of tumour-infiltrating lymphocyte cell therapy for advanced melanoma: a systematic review
Source: BMC Cancer. 2026 Mar 20;26:538. doi: 10.1186/s12885-026-15888-5 (PMC13127032; doi:10.1186/s12885-026-15888-5)
Supplement: Supplementary file 1 — Supplementary Material 1. [file 12885_2026_15888_MOESM1_ESM.zip › 2-Supplementary Table 1.docx]

Supplementary Table 1. Search strategy for EMBASE

| No. | Search Strategy | Number of papers |
| --- | --- | --- |
| 1. | economic evaluation/ or “cost effectiveness analysis”/ or “cost utility analysis”/ or “cost benefit analysis”/ | 323,172 |
| 2. | (economic evaluation or cost effectiveness or cost utility or cost benefit).ab. | 130,750 |
| 3. | 1 or 2 | 349,739 |
| 4. | tumor associated leukocyte/ | 39,682 |
| 5. | tumo?r infiltrating lymphocyte.ab. | 1,466 |
| 6. | 4 or 5 | 39,902 |
| 7. | melanoma | 166,662 |
| 8. | (stage IIIC or IV melanoma or metastatic melanoma or unresectable melanoma).ab. | 23,189 |
| 9. | 7 or 8 | 176,362 |
| 10. | 3 and 6 and 9 | 26 |
