# Supplementary material for: The cost-effectiveness of tumour-infiltrating lymphocyte cell therapy for advanced melanoma: a systematic review
Source: BMC Cancer. 2026 Mar 20;26:538. doi: 10.1186/s12885-026-15888-5 (PMC13127032; doi:10.1186/s12885-026-15888-5)
Supplement: Supplementary file 1 — Supplementary Material 1. [file 12885_2026_15888_MOESM1_ESM.zip › 2-Supplementary Table 2.docx]

Supplementary Table 2. Quality assessment result of included studies using the QHES

|  |  | Retal et al. 2018 | | ten Ham et al. 2024 | |
| --- | --- | --- | --- | --- | --- |
| Questions | Points | Yes/No (Y/N) | | Yes/No (Y/N) | |
| Q1. Study objective | 7 | Y | 7 | Y | 7 |
| Q2. Perspective | 4 | Y | 4 | Y | 4 |
| Q3. Variables source | 8 | Y | 8 | Y | 8 |
| Q4. Subgroup analysis | 1 | Y | 1 | Y | 1 |
| Q5. Uncertainty | 9 | Y | 9 | Y | 9 |
| Q6. Incremental analysis | 6 | Y | 6 | Y | 6 |
| Q7. Data abstraction | 5 | Y | 5 | Y | 5 |
| Q8. Time horizon + discounting | 7 | Y | 7 | Y | 7 |
| Q9. Cost measurement | 8 | Y | 8 | Y | 8 |
| Q10. Primary outcome | 6 | Y | 6 | Y | 6 |
| Q11. Valid outcome measures | 7 | Y | 7 | Y | 7 |
| Q12. Transparent model structure | 8 | Y | 8 | Y | 8 |
| Q13. Model choice & assumptions | 7 | Y | 7 | Y | 7 |
| Q14. Biases | 6 | Y | 6 | Y | 6 |
| Q15. Conclusions justified | 8 | Y | 8 | Y | 8 |
| Q16. Source of funding | 3 | N | 0 | Y | 3 |
| Total points | 100 |  | 97 |  | 100 |
